# Time course of cardiac rupture after acute myocardial infarction and comparison of clinical features of different rupture types

**DOI:** 10.3389/fcvm.2024.1365092

**Published:** 2024-04-09

**Authors:** Chendi Liang, Xiaoxia Wang, Peng Yang, Ru Zhao, Li Li, Zhixin Wang, Yanqing Guo

**Affiliations:** ^1^ Department of Cardiology, Shanxi Cardiovascular Hospital, Taiyuan, Shanxi, China; ^2^ Department of Medical Oncology, Beijing YouAn Hospital, Capital Medical University, Beijing, China; ^3^ Precision Laboratory of Vascular Medicine, Shanxi Cardiovascular Hospital, Taiyuan, Shanxi, China

**Keywords:** cardiac rupture, acute myocardial infarction, CR, AMI, time course, clinical features

## Abstract

**Objective:**

This study aimed to investigate the time course of cardiac rupture (CR) after acute myocardial infarction (AMI) and the differences among different rupture types.

**Method:**

We retrospectively analyzed 145 patients with CR after AMI at Shanxi Cardiovascular Hospital from June 2016 to September 2022. Firstly, according to the time from onset of chest pain to CR, the patients were divided into early CR (≤24 h) (*n* = 61 patients) and late CR (>24 h) (*n* = 75 patients) to explore the difference between early CR and late CR. Secondly, according to the type of CR, the patients were divided into free wall rupture (FWR) (*n* = 55) and ventricular septal rupture (VSR) (*n* = 90) to explore the difference between FWR and VSR.

**Results:**

Multivariate logistic regression analysis showed that high white blood cell count (OR = 1.134, 95% CI: 1.019–1.260, *P* = 0.021), low creatinine (OR = 0.991, 95% CI: 0.982–0.999, *P* = 0.026) were independently associated with early CR. In addition, rapid heart rate (OR = 1.035, 95% CI: 1.009–1.061, *P* = 0.009), low systolic blood pressure (OR = 0.981, 95% CI: 0.962–1.000, *P* = 0.048), and anterior myocardial infarction (OR = 5.989, 95% CI: 1.978–18.136, *P* = 0.002) were independently associated with VSR.

**Conclusion:**

In patients with CR, high white blood cell count and low creatinine were independently associated with early CR, rapid heart rate, low systolic blood pressure, and anterior myocardial infarction were independently associated with VSR.

## 1 Introduction

Cardiac rupture (CR) is a rare but severe complication of acute myocardial infarction (AMI), including free wall rupture (FWR), ventricular septal rupture (VSR), and papillary muscle rupture. With the advancement of reperfusion therapies (especially percutaneous coronary intervention), the mortality and complication rate of AMI have dropped remarkably. However, the mortality caused by CR remains high (1–3). As a rare complication, although most studies have investigated the risk factors of CR after AMI, but the time course of CR and the differences among different rupture types are still unknown. Therefore, we analyzed the data of CR patients from the Shanxi Cardiovascular Hospital to explore the time course of CR and the differences among different rupture types.

## 2 Materials and methods

We retrospectively analyzed 145 patients with CR after AMI from Shanxi Cardiovascular Hospital between June 2016 and September 2022, including 55 patients with FWR and 90 patients with VSR. The study was approved by the ethics committee of Shanxi Cardiovascular Hospital.

### 2.1 Definition

AMI was defined as the presence of clinical evidence of acute myocardial injury with acute myocardial ischaemia, with detectable increase and/or decrease in cardiac troponin, which at least one value above the 99th percentile of the upper reference limit, and at least one of the following: (1) Symptoms of acute myocardial ischemia; (2) New ischemic electrocardiographic changes; (3) Development of pathogenic Q waves; (4) Imaging evidence of new loss of viable myocardium or new regional wall motion abnormality; and (5) Coronary thrombus confirmed by coronary angiography or intracavitary imaging, or autopsy (4).

The diagnosis of FWR was based on sudden cardiogenic shock or hypotension and echocardiography indicating a large pericardial effusion. The diagnosis of VSR was based on an emerging systolic murmur, and echocardiography confirms a shunt at the ventricular level.

Early CR was defined as the time from onset of chest pain to CR ≤ 24 h. Late CR was defined as the time from onset of chest pain to CR > 24 h.

### 2.2 Data collection

We extracted the following data from the medical records: basic clinical characteristics of the patients, medical history, time from onset of chest pain to admission, vital signs at admission, haematological parameters, etc.

### 2.3 Statistical analysis

SPSS 19.0 software was used for statistical analysis. Normally distributed continuous variables were expressed using mean ± standard deviation and analyzed using a *t*-test. Non-normally distributed continuous variables were expressed using the median (interquartile range) and analyzed using the Mann–Whitney *U*-test. Dichotomous variables were expressed by percentage and analyzed using chi-square tests. Multivariate logistic regression models were constructed using the above variables with *P* < 0.1 to evaluate the differences between early CR and late CR, FWR and VSR. A two-sided *P* < 0.05 was considered statistically different.

## 3 Results

### 3.1 Time course of CR

**Figure 1:**
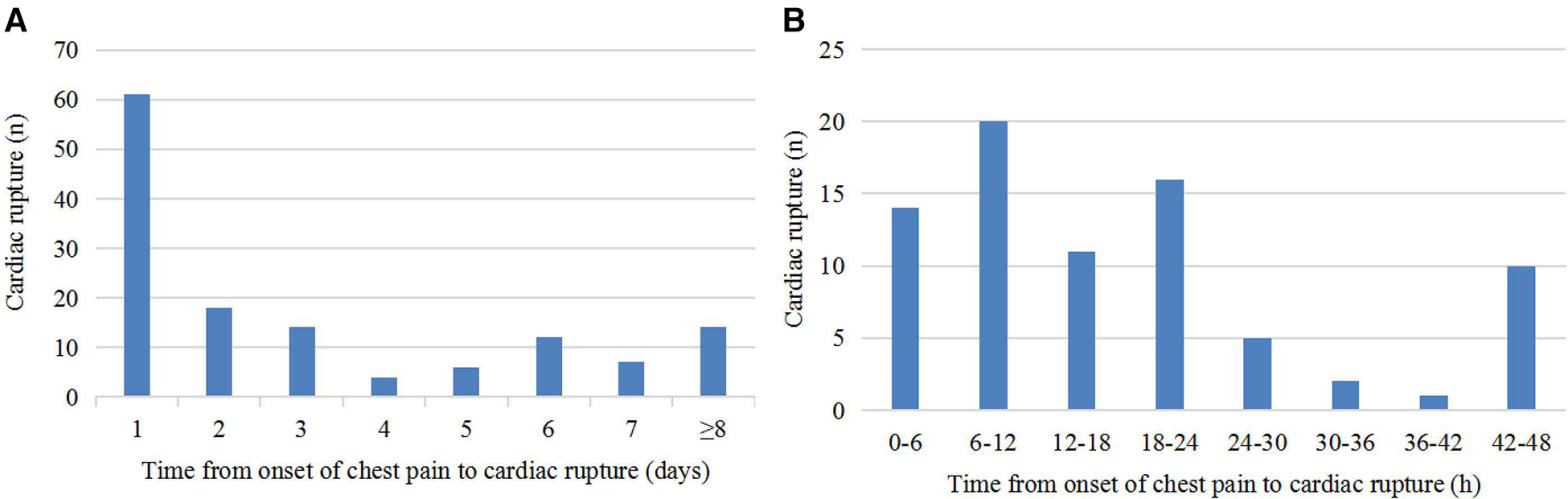
Time course of CR. (**A**) Time from onset of chest pain to CR. (**B**) Distribution of patients with CR within 48 h after onset of chest pain.

Of the 145 patients with CR after AMI, 136 patients had detailed records of the time from onset of chest pain to CR. As shown in Figure 1A, CR occurred to 61 (44.9%) patients within 24 h of chest pain onset, 79 (58.1%) patients within the first 48 h, and 122 (89.7%) patients within the first week. As shown in Figure 1B, 6–12 h after chest pain onset is the peak time of CR occurrence.

### 3.2 Comparison of early CR and late CR

Of the 145 patients with CR after AMI, 136 patients had detailed records of the time from onset of chest pain to CR, including 61 patients with early CR (≤24 h) and 75 patients with late CR (>24 h). The clinical characteristics of patients with early rupture and late rupture are shown in Table 1. Compared with the early CR group, the late CR group had a higher proportion of males and smokers. In terms of medications, P2Y12 receptor antagonists and statins were used more frequently in the late CR group. In addition, the incidence of abnormal Q waves was higher in the late CR group, while the white blood cell count in the late CR was significantly lower than in the early CR group. After adjusting for potential confounders, multivariate logistic regression analysis showed that high white blood cell count (OR = 1.134, 95% CI: 1.019–1.260, *P* = 0.021) and low creatinine (OR = 0.991, 95% CI: 0.982–0.999, *P* = 0.026) were independently associated with early CR (see Table 2).

**Table 1:** Comparison of clinical characteristics between early rupture and late rupture.

Variables	Early rupture (*n* = 61)	Late rupture (*n* = 75)	*P*-value
Age, years	71.61 ± 10.37	69.8 ± 9.538	0.293
Male, *n* (%)	22 (36.1%)	42 (56%)	0.021
Hypertensive, *n* (%)	38 (62.3%)	43 (57.3%)	0.558
Diabetes, *n* (%)	18 (29.5%)	17 (22.7%)	0.364
Smoking, *n* (%)	14 (23%)	33 (44%)	0.01
Alcohol, *n* (%)	9 (14.8%)	14 (18.7%)	0.545
History of angina/myocardial infarction	8 (13.1%)	13 (17.3%)	0.498
Heart rate (bpm)	90.44 ± 21.691	92.27 ± 20.297	0.614
Systolic blood pressure (mmHg)	113.51 ± 30.820	112.18 ± 20.691	0.773
Diastolic blood pressure (mmHg)	70.43 ± 20.162	67.99 ± 13.915	0.424
Abnormal Q wave	48 (78.7%)	69 (92%)	0.026
Infarct location, *n* (%)			
Anterior	45 (73.8%)	44 (58.7%)	0.065
Inferior	19 (31.1%)	32 (42.7%)	0.168
Posterior	7 (11.5%)	7 (9.3%)	0.683
Lateral	2 (3.3%)	1 (1.3%)	0.856
Multiple site	13 (21.3%)	15 (20%)	0.851
Laboratory values at admission			
White blood cell (×10^9^/L)	12.4 (10.65, 14.54)	11.69 (8.4, 14.7)	0.040
Red blood cell (×10^9^/L)	4.09 (3.71, 4.34)	4.11 (3.85, 4.43)	0.414
Platelet (×10^9^/L)	202.98 ± 64.54	214.82 ± 72.66	0.878
Creatinine (umol/L)	78.8 (59.25, 92.2)	89.8 (63.2, 117.6)	0.067
Blood urea nitrogen (mmol/L)	8.9 (6.72, 11.33)	8.8 (6.9, 12.1)	0.500
Medication			
Aspirin, *n* (%)	43 (70.5%)	57 (76%)	0.469
P2Y12 receptor antagonist, *n* (%)	45 (73.8%)	70 (93.3%)	0.002
Statin, *n* (%)	38 (62.3%)	63 (84%)	0.004
Cardiac rupture			
Free wall rupture, *n* (%)	26 (42.6%)	29 (38.7%)	0.64
Ventricular septal rupture, *n* (%)	35 (57.4%)	46 (61.3%)	0.64

**Table 2:** Multivariate logistic regression analysis of early rupture.

Variables	*β*	OR	95% CI	*P*-value
Male	−0.099	0.905	0.339–2.419	0.843
Smoking	−0.199	0.819	0.296–2.268	0.701
Anterior	0.696	2.005	0.832–4.832	0.121
White blood cell	0.125	1.134	1.019–1.260	0.021
Creatinine	−0.01	0.991	0.982–0.999	0.026
P2Y12 receptor antagonist	−0.991	0.371	0.107–1.291	0.119
Statin	−0.790	0.454	0.168–1.229	0.120
Abnormal Q wave	−0.609	0.544	0.155–1.915	0.343

### 3.3 Comparison of FWR and VSR

Of the 145 patients with CR after AMI, including 55 patients with FWR and 90 patients with VSR. The clinical characteristics of patients with FWR and VSR are shown in Table 3. Compared with the FWR group, the VSR group had faster heart rates, lower systolic blood pressure and higher urea nitrogen levels. In terms of medications, statins were used more frequently in the VSR group. In addition, anterior wall myocardial infarction was more frequent in the VSR patients, while the FWR patients presented more frequently with inferior wall myocardial infarction and posterior wall myocardial infarction. After adjusting for potential confounders, multivariate logistic regression analysis showed that rapid heart rate (OR = 1.035, 95% CI: 1.009–1.061, *P* = 0.009), low systolic blood pressure (OR = 0.981, 95% CI: 0.962–1.000, *P* = 0.048), anterior wall myocardial infarction (OR = 5.989, the 95% CI: 1.978–18.136, *P* = 0.002) were independently associated with VSR (see Table 4).

**Table 3:** Comparison of clinical characteristics between FWR and VSR.

Variables	FWR (*n* = 55)	VSR (*n* = 90)	*P*-value
Age, years	71.62 ± 10.31	69.34 ± 9.7	0.183
Male, *n* (%)	31 (51.4%)	39 (43.3%)	0.128
Hypertensive, *n* (%)	32 (58.2%)	55 (61.1%)	0.727
Diabetes, *n* (%)	12 (21.8%)	24 (26.7%)	0.512
Smoking, *n* (%)	16 (29.1%)	35 (38.9%)	0.231
Alcohol, *n* (%)	10 (18.2%)	17 (18.9%)	0.915
History of angina/myocardial infarction	8 (14.5%)	15 (16.7%)	0.734
Heart rate (bpm)	76 (66, 94)	98 (85.75, 109.25)	<0.001
Systolic blood pressure (mmHg)	118.51 ± 24.82	109.58 ± 24.66	0.037
Diastolic blood pressure (mmHg)	71.60 ± 15.43	67.37 ± 17.43	0.141
Abnormal Q wave	44 (80%)	80 (88.9%)	0.140
Infarct location, *n* (%)			
Anterior	21 (38.2%)	75 (83.3%)	<0.001
Inferior	27 (49.1%)	24 (26.7%)	0.006
Posterior	12 (21.8%)	2 (2.2%)	<0.001
Lateral	3 (5.5%)	0 (0.0%)	0.101
Multiple site	12 (21.8%)	16 (17.8%)	0.550
Laboratory values at admission			
White blood cell (×10^9^/L)	12.3 (9.3, 14.6)	11.65 (9.27, 14.25)	0.321
Red blood cell (×10^9^/L)	4.15 (4.03, 4.41)	4.06 (3.69, 4.37)	0.293
Platelet (×10^9^/L)	192.87 (149, 231)	211 (159.75, 266.25)	0.096
Creatinine (umol/L)	87.16 (63.8, 96.5)	81.65 (58.55, 118.95)	0.667
Blood urea nitrogen (mmol/L)	8.6 (6.4, 9.3)	8.65 (7.30, 11.95)	0.028
Medication			
Aspirin, *n* (%)	43 (78.2%)	65 (72.2%)	0.424
P2Y12 receptor antagonist, *n* (%)	43 (78.2%)	80 (88.9%)	0.081
Statin, *n* (%)	36 (65.5%)	73 (81.1%)	0.034

**Table 4:** Multivariate logistic regression analysis of ventricular septal rupture.

Variables	*β*	OR	95% CI	*P*-value
Heart rate	0.034	1.035	1.009–1.061	0.009
Systolic blood pressure	−0.020	0.981	0.962–1.000	0.048
Anterior	1.790	5.989	1.978–18.136	0.002
Inferior	0.324	1.383	0.453–4.227	0.569
Posterior	−1.901	0.149	0.021–1.079	0.059
Platelet	0.005	1.005	0.999–1.012	0.096
Blood urea nitrogen	0.078	1.082	0.979–1.195	0.123
P2Y12 receptor antagonist	0.850	2.340	0.658–8.323	0.189
Statin	0.914	2.495	0.803–7.753	0.114

## 4 Discussion

CR is a rare but severe life-threatening complication of AMI. In the pre-reperfusion era, the incidence of CR after AMI was as high as 6.2%. With the development of reperfusion technology, especially the widespread use of percutaneous coronary intervention (PCI), the incidence of CR after AMI has been significantly reduced (5). Elbadawi et al. (2) enrolled 3,951,861 patients with ST-segment elevation myocardial infarction (STEMI) and 5,114,270 patients with non-ST-segment elevation myocardial infarction (NSTEMI) between 2003 and September 2015, and the incidence of CR was 0.27% and 0.06%, respectively. A multicentre study from Japan (6) found that as the proportion of PCI increased, the incidence of CR decreased from 3.7% between 1997 and 2004 to 1.9% between 2011 and 2014. Although the incidence of CR after AMI has declined, the death rate of CR can still be as high as 50% (1).

In this study, we selected patients with CR after AMI and attempted to compare the clinical characteristics of early CR and late CR, FWR and VSR. Multivariate logistic regression analysis showed that high white blood cell count (OR = 1.134, 95% CI: 1.019–1.260, *P* = 0.021), low creatinine (OR = 0.991, 95% CI: 0.982–0.999, *P* = 0.026) were independently associated with early CR, rapid heart rate (OR = 1.035, 95% CI: 1.009–1.061, *P* = 0.009), low systolic blood pressure (OR = 0.981, 95% CI: 0.962–1.000, *P* = 0.048), and anterior myocardial infarction (OR = 5.989, 95% CI: 1.978–18.136, *P* = 0.002) were independently associated with VSR.

FWR refers to the rupture of the left ventricular wall other than the interventricular septum, including the anterior, lateral, and posterior walls of the left ventricle. Rupture is common in the anterior and lateral walls of the anterior descending branch distribution, is more frequent in the apical region, and often occurs at the junction of normal and infarcted myocardium. VSR is caused by occlusion of the left anterior descending branch, the dominant right coronary artery, or the dominant left circumflex branch. VSR caused by anterior wall myocardial infarction is more often near the apex of the heart, and that caused by inferior wall myocardial infarction is at the base of the interventricular septum. As far as we know, cardiac rupture usually occurs within first week after AMI, especially within the first 24 h and 3–5 days (7), which means that the mechanism of early CR may be different from that of late CR. Becker and van Mantgem (8) categorized FWR into three types: Type I rupture is characterized by a sudden slit-like rupture that usually occurs in the acute phase of AMI (<24 h); Type II rupture is an erosive rupture, which usually occurs 24 h after AMI and is caused by erosion and chronic tearing of the infarct site; and type III rupture usually occurs in the late phase of AMI (which usually occurs more than one week later), and is a ventricular wall rupture due to overexpansion of the ventricular aneurysm. For early CR, AMI resulted in massive apoptosis of cardiomyocytes in the infarction area and decreased myocardial contractility and ventricular wall compliance. The infarct area showed increased brittleness compared to the non-infarct area. Therefore, when the myocardium contracts and diastoles, the non-infarcted area will form a shear effect with the infarct area, resulting in the occurrence of cardiac rupture (9). For advanced CR, a massive aggregate infiltration of inflammatory cells into the infarct zone begins 2–3 days after AMI with a peak around 1 week (10), among which white blood cells can release matrix metalloproteinases (MMPs). MMPs, a family of structurally similar zinc-dependent endopeptidases, can promote the degradation of extracellular matrix, activate cytokines and other MMP precursors, and play a key role in tissue remodeling (11). In the early stage of myocardial infarction, the massive infiltration of inflammatory cells will promote the activation of MMPs, leading to the massive degradation of the extracellular matrix such as myocardial collagen, fibronectin, and laminin, resulting in the loss of myocardial mesenchymal skeleton support and protection, and the disruption of the tissue structure, promoting disproportionate thinning and expansion of the infarcted areas and ultimately leading to CR (12, 13). In addition, animal studies have shown that myocardial tensile strength begins to decline within 24 h after the onset of AMI, and it can be reduced by 60% during the peak period (3–4 days) (14). Gong et al. (15) found that 6–24 h after STEMI was the peak period of FWR by analyzing 78 patients with FWR after STEMI. In addition, Shoji et al. (16) found that within 24 h and 6 days after AMI was the peak period of CR. This paper also confirms that 6–12 h after AMI is the peak time for the occurrence of CR.

Leukocytes play a critical role in the development of AMI. It has been shown that high leukocyte levels after AMI are closely associated with higher mortality, severe ventricular arrhythmias, cardiogenic shock, and acute heart failure (17). Dharma et al. (18) analyzed 585 patients with NSTEMI and found that high leukocyte levels (>11,000/μl) increased the incidence of major adverse cardiovascular events (MACE). In addition, lowering the white blood cell count significantly reduced the inflammatory response and improved cardiac function in rats with AMI (19). Qian et al. (20) found that AMI patients with elevated white blood cells were more likely to suffer CR. Our research showed that high white blood cell count was closely related to early CR (≤24 h), which may be related to the massive infiltration of white blood cells and the massive expression of MMP-9 in the early stage of myocardial infarction resulting in structural changes in the myocardial extracellular matrix. Renal dysfunction is considered a risk factor for cardiovascular disease and is strongly associated with poor prognosis of coronary heart disease (21). We found that patients with early CR (≤24 h) had lower creatinine levels. The effect of renal dysfunction on the occurrence time of CR after AMI still needs further study.

AMI is usually accompanied by sympathetic activation, which is mainly manifested by massive secretion of catecholamines, antidiuretic hormones, and increased activity of the renin-angiotensin-aldosterone system, resulting in increased cardiac afterload and rapid heart rate (22). The rapid heart rate will frequently pull the myocardium in the infarcted area and contribute to the occurrence of CR. It has been reported that for every 30 bpm increase in heart rate, there is a 1.32 times increase in the risk of CR (23). Early use of beta-blockers can reduce the risk of CR, and this beneficial effect is closely associated with the decrease in heart rate (24). A meta-analysis including four randomized controlled trials found that early use of beta-blockers reduced the incidence of CR after AMI by 32% (25). However, hypertension was recognized as a risk factor for CR in the past few decades, but due to recent changes in the definition of hypertension and the use of antihypertensive medications, hypertension is no longer considered to be significantly associated with CR (1). It is currently believed that good blood pressure reflects better ventricular function, and higher systolic blood pressure on admission is considered to be an important marker of good prognosis for CR (26). Anterior myocardial infarction is considered a risk factor for CR (1), and VSD is more common in anterior wall STEMI (2). The anterior upper 2/3 of the ventricular septum is supplied by the anterior descending branch, whereas the posterior lower 1/3 is supplied by the posterior descending branch. Acute lesions of the anterior descending branch can cause paradoxical movement of the infarcted ventricular septum during the systolic period, thus contributing to the development of VSR, which is more common in complete occlusion of the anterior descending branch (27). In this study, we found that rapid heart rate, low systolic blood pressure, and anterior myocardial infarction were strongly associated with VSR.

As we all know, primary PCI can reduce the risk of CR after AMI, whereas thrombolysis can increase this trend. Bueno et al. (28) analyzed 706 patients with AMI and found that the incidence of FWR was higher in patients treated with thrombolytic therapy (17.1%) than in patients who did not receive reperfusion therapy (7.9%) or who underwent PCI (4.9%) (*P* < 0.0001). However, it should be noted that the timing of thrombolytic therapy is closely related to the risk of CR. A meta-analysis (29) including 1,638 patients found that the risk of CR was 0.4 (95% CI: 0.17–0.93) for patients who received thrombolytic therapy at 7 h after AMI, 0.93 (95% CI: 0.53–1.6) at 11 h, and 3.21 (95% CI: 1.10–10.1) at 17 h. It indicates that thrombolytic therapy in the early stage of AMI can reduce the risk of CR, while late thrombolytic therapy can increase the risk of CR. In addition, different treatment strategies reveal different pathologic mechanisms of CR. Honda et al. (1) found a high incidence of myocardial hemorrhage in the infarcted area in 63 patients with CR who treated with PCI or thrombolysis. Becker type 1 and 2 ruptures were common in patients who received reperfusion therapy, whereas Becker type 3 rupture was common in patients who did not receive reperfusion therapy. However, because this study was retrospective and limited by clinical data, the number of thrombolysis and PCI patients were not counted. Further attention will be paid to this aspect in subsequent clinical studies.

There are some limitations in this study. Firstly, as a retrospective study, some information was unavailable or incomplete. In addition, due to the low incidence of CR, the sample size included in this study was small, and there were some errors and limitations in stating the conclusions. Therefore, multicenter studies are still needed for further evaluation.

## 5 Conclusion

In patients with CR, high white blood cell count and low creatinine were independently associated with early CR, rapid heart rate, low systolic blood pressure, and anterior myocardial infarction were independently associated with VSR.

## Data Availability

The raw data supporting the conclusions of this article will be made available by the authors, without undue reservation.

## References

[1] Honda S Asaumi Y Yamane T Nagai T Miyagi T Noguchi T Trends in the clinical and pathological characteristics of cardiac rupture in patients with acute myocardial infarction over 35 years. J Am Heart Assoc. (2014) 3(5):e000984. 10.1161/JAHA.114.000984 25332178 PMC4323797

[2] Elbadawi A Elgendy IY Mahmoud K Barakat AF Mentias A Mohamed AH Temporal trends and outcomes of mechanical complications in patients with acute myocardial infarction. JACC Cardiovasc Interv. (2019) 12(18):1825–36. 10.1016/j.jcin.2019.04.039 31537282

[3] Puerto E Viana-Tejedor A Martínez-Sellés M Domínguez-Pérez L Moreno G Martín-Asenjo R Temporal trends in mechanical complications of acute myocardial infarction in the elderly. J Am Coll Cardiol. (2018) 72(9):959–66. 10.1016/j.jacc.2018.06.031 30139440

[4] Thygesen K Alpert JS Jaffe AS Chaitman BR Bax JJ Morrow DA Fourth universal definition of myocardial infarction (2018). J Am Coll Cardiol. (2018) 72(18):2231–64. 10.1016/j.jacc.2018.08.1038 30153967

[5] Figueras J Alcalde O Barrabés JA Serra V Alguersuari J Cortadellas J Changes in hospital mortality rates in 425 patients with acute ST-elevation myocardial infarction and cardiac rupture over a 30-year period. Circulation. (2008) 118(25):2783–9. 10.1161/CIRCULATIONAHA.108.776690 19064683

[6] Koeda Y Itoh T Ishikawa Y Morino Y Mizutani T Ako J A multicenter study on the clinical characteristics and risk factors of in-hospital mortality in patients with mechanical complications following acute myocardial infarction. Heart Vessels. (2020) 35(8):1060–9. 10.1007/s00380-020-01586-0 32239276

[7] Birnbaum Y Fishbein MC Blanche C Siegel RJ. Ventricular septal rupture after acute myocardial infarction. N Engl J Med. (2002) 347(18):1426–32. 10.1056/NEJMra020228 12409546

[8] Becker AE van Mantgem JP. Cardiac tamponade. A study of 50 hearts. Eur J Cardiol. (1975) 3(4):349–58. 1193118

[9] Matsusaka H Ide T Matsushima S Ikeuchi M Kubota T Sunagawa K Targeted deletion of p53 prevents cardiac rupture after myocardial infarction in mice. Cardiovasc Res. (2006) 70(3):457–65. 10.1016/j.cardiores.2006.02.001 16533502

[10] Zidar N Jeruc J Balazic J Stajer D. Neutrophils in human myocardial infarction with rupture of the free wall. Cardiovasc Pathol. (2005) 14(5):247–50. 10.1016/j.carpath.2005.04.002 16168897

[11] Bräuninger H Krüger S Bacmeister L Nyström A Eyerich K Westermann D Matrix metalloproteinases in coronary artery disease and myocardial infarction. Basic Res Cardiol. (2023) 118(1):18. 10.1007/s00395-023-00987-2 37160529 PMC10169894

[12] Becirovic-Agic M Chalise U Daseke MJ II Konfrst S Salomon JD Mishra PK Infarct in the heart: what’s MMP-9 got to do with it? Biomolecules. (2021) 11(4):491. 10.3390/biom11040491 33805901 PMC8064345

[13] Deleon-Pennell KY Altara R Yabluchanskiy A Modesti A Lindsey ML. The circular relationship between matrix metalloproteinase-9 and inflammation following myocardial infarction. IUBMB Life. (2015) 67(8):611–8. 10.1002/iub.1408 26269290 PMC4553095

[14] Gao XM Xu Q Kiriazis H Dart AM Du XJ. Mouse model of post-infarct ventricular rupture: time course, strain- and gender-dependency, tensile strength, and histopathology. Cardiovasc Res. (2005) 65(2):469–77. 10.1016/j.cardiores.2004.10.014 15639486

[15] Gong W Shi H Yan M Yan Y Wang X Li S Clinical manifestation, timing course, precipitating factors, and protective factors of ventricular free wall rupture following ST-segment elevation myocardial infarction. Int Heart J. (2020) 61(4):651–7. 10.1536/ihj.19-541 32684590

[16] Shoji K Yanishi K Kawamata H Hori Y Fujioka A Kohno Y New risk factors for early- and late-onset cardiac rupture in ST-elevation myocardial infarction patients after primary percutaneous coronary intervention. J Cardiol. (2022) 79(3):400–7. 10.1016/j.jjcc.2021.10.006 34696926

[17] Ghaffari S Nadiri M Pourafkari L Sepehrvand N Movasagpoor A Rahmatvand N The predictive value of total neutrophil count and neutrophil/lymphocyte ratio in predicting in-hospital mortality and complications after STEMI. J Cardiovasc Thorac Res. (2014) 6(1):35–41. 10.5681/jcvtr.2014.007 24753830 PMC3992730

[18] Dharma S Hapsari R Siswanto BB van der Laarse A Jukema JW. Blood leukocyte count on admission predicts cardiovascular events in patients with acute non-ST elevation myocardial infarction. Int J Angiol. (2015) 24(2):127–32. 10.1055/s-0035-1544178 26060384 PMC4452600

[19] Zhu G Yao Y Pan L Zhu W Yan S. Reduction of leukocyte counts by hydroxyurea improves cardiac function in rats with acute myocardial infarction. Med Sci Monit. (2015) 21:3941–7. 10.12659/msm.893744 26675565 PMC4687945

[20] Qian G Wu C Chen YD Tu CC Wang JW Qian YA. Predictive factors of cardiac rupture in patients with ST-elevation myocardial infarction. J Zhejiang Univ Sci B. (2014) 15(12):1048–54. 10.1631/jzus.B1400095 25471834 PMC4265559

[21] Tsai IT Wang CP Lu YC Hung WC Wu CC Lu LF The burden of major adverse cardiac events in patients with coronary artery disease. BMC Cardiovasc Disord. (2017) 17(1):1. 10.1186/s12872-016-0436-7 28052754 PMC5210314

[22] McAlpine HM Morton JJ Leckie B Rumley A Gillen G Dargie HJ. Neuroendocrine activation after acute myocardial infarction. Br Heart J. (1988) 60(2):117–24. 10.1136/hrt.60.2.117 3415870 PMC1216532

[23] López-Sendón J Gurfinkel EP Lopez de Sa E Agnelli G Gore JM Steg PG Factors related to heart rupture in acute coronary syndromes in the global registry of acute coronary events. Eur Heart J. (2010) 31(12):1449–56. 10.1093/eurheartj/ehq061 20231153

[24] Fox K Borer JS Camm AJ Danchin N Ferrari R Lopez Sendon JL Resting heart rate in cardiovascular disease. J Am Coll Cardiol. (2007) 50(9):823–30. 10.1016/j.jacc.2007.04.079 17719466

[25] Gong W Feng S Wang X Fan J Li A Nie SP. Beta-blockers reduced the risk of cardiac rupture in patients with acute myocardial infarction: a meta-analysis of randomized control trials. Int J Cardiol. (2017) 232:171–5. 10.1016/j.ijcard.2017.01.035 28109576

[26] Sulzgruber P El-Hamid F Koller L Forster S Goliasch G Wojta J Long-term outcome and risk prediction in patients suffering acute myocardial infarction complicated by post-infarction cardiac rupture. Int J Cardiol. (2017) 227:399–403. 10.1016/j.ijcard.2016.11.037 27847155

[27] Yip HK Fang CY Tsai KT Chang HW Yeh KH Fu M The potential impact of primary percutaneous coronary intervention on ventricular septal rupture complicating acute myocardial infarction. Chest. (2004) 125(5):1622–8. 10.1378/chest.125.5.1622 15136368

[28] Bueno H Martínez-Sellés M Pérez-David E López-Palop R. Effect of thrombolytic therapy on the risk of cardiac rupture and mortality in older patients with first acute myocardial infarction. Eur Heart J. (2005) 26(17):1705–11. 10.1093/eurheartj/ehi284 15855190

[29] Honan MB Harrell HF Jr Reimer KA Califf RM Mark DB Pryor DB Cardiac rupture, mortality and the timing of thrombolytic therapy: a meta-analysis. J Am Coll Cardiol. (1990) 16(2):359–67. 10.1016/0735-1097(90)90586-e 2142705

